# Synthesis, Structure, Surface and Antimicrobial Properties of New Oligomeric Quaternary Ammonium Salts with Aromatic Spacers

**DOI:** 10.3390/molecules22111810

**Published:** 2017-10-25

**Authors:** Bogumił Brycki, Anna Koziróg, Iwona Kowalczyk, Tomasz Pospieszny, Paulina Materna, Jędrzej Marciniak

**Affiliations:** 1 Laboratory of Microbiocides Chemistry, Faculty of Chemistry, Adam Mickiewicz University in Poznań, Umultowska 89b, 61-614 Poznań, Poland; iwkow@amu.edu.pl (I.K.); tposp@amu.edu.pl (T.P.); p_materna@wp.pl (P.M.); 2 Institute of Fermentation Technology and Microbiology, Faculty of Biotechnology and Food Sciences, Lodz University of Technology, Wólczańska 171/173, 90-924 Łódź, Poland; anna.kozirog@p.lodz.pl; 3 Department of Materials Chemistry, Faculty of Chemistry, Adam Mickiewicz University in Poznań, Umultowska 89b, 61-614 Poznań, Poland; jedrzej.marciniak@amu.edu.pl

**Keywords:** quaternary ammonium salts, oligomeric surfactants, rigid spacer, spectroscopy, synthesis, CMC, antimicrobial properties

## Abstract

New dimeric, trimeric and tetrameric quaternary ammonium salts were accomplished by reaction of tertiary alkyldimethyl amines with appropriate bromomethylbenzene derivatives. A series of new cationic surfactants contain different alkyl chain lengths (C4–C18), aromatic spacers and different numbers of quaternary nitrogen atoms. The structure of the products was confirmed by spectral analysis (FT-IR, ^1^H-NMR, ^13^C-NMR and 2D-NMR), mass spectroscopy (ESI-MS), elemental analysis, as well as PM5 semiempirical methods. Compound (**21**) was also analyzed using X-ray crystallography. Critical micelle concentration (CMC) of 1,4-bis-[*N*-(1-alkyl)-*N*,*N*-dimethylammoniummethyl]benzene dibromides (**3**–**9**) was determined to characterize the aggregation behavior. The antimicrobial properties of novel QACs (Quaternary Ammonium Salts) were examined to set their minimal inhibitory concentration (MIC) values against fungi *Aspergillus niger*, *Candida albicans*, *Penicillium chrysogenum* and bacteria *Staphylococcus aureus*, *Bacillus subtilis*, *Escherichia coli* and *Pseudomonas aeruginosa*.

## 1. Introduction

Anionic, cationic, non-ionic and amphoteric surfactants form one of the most useful groups of chemicals. The hydrophilic-hydrophobic balance (HLB) of these amphiphilic compounds is a crucial key for their applications in detergents [1], personal care products [2], paint and coatings additives [3], natural and synthetic dyes [4,5], microbiocides [6,7,8], organic synthesis [9,10], pharmacy [4,11], textiles, leather and paper [1,12,13], agrochemicals [14], fibers [15,16,17], plastics [18], food processing [19,20], enhanced oil recovery [21,22,23], environmental protection (*oil slick dispersant*) [24,25], and explosives [26]. Surfactants also play a significant role in the development of high technologies and preparation of nano- and smart materials [27]. The global surfactant market exceeded 15 million tons in 2015 and is still growing up a 4.20% CAGR (*Compound Annual Growth Rate*) [28]. The growing demand on surfactants is mainly driven by personal care products and detergents (anionics and nonionics) as well as anticorrosion and biocidal products (cationics). To avoid disadvantageous impacts of such huge amounts of surfactants on the environment, a new type of more effective amphiphiles should be investigated and applied in the near future. One of the promising ways is Gemini surfactants. These compounds contain two hydrophilic head groups and two hydrophobic tails linked by a spacer at the head groups or close to them. The spacer can be either hydrophobic (polymethylene chain), or hydrophilic (polymethylene chain with oxygen or nitrogen atoms). From a structural point of view a spacer can be rigid (aromatic or unsaturated linear hydrocarbons) or flexible (polymethylene chain). The neutral charge of the cationic Gemini surfactants is retained by the presence of counterions, which can be organic or inorganic. The Gemini alkylammonium salts show a unique surface and interfacial properties in aqueous solution. Critical micelle concentrations (CMC) of Gemini surfactants are usually much lower, even up to 100 times in comparison to CMCs of the corresponding monomeric surfactants. The effectiveness of dimeric surfactants in lowering the surface tension is also much better than their monomeric analogs. The values of C_20_, i.e., surfactant concentration at which the surface tension (γ) is lowered by 20 mN/m, are a dozen times smaller for Gemini surfactants in comparison to monomeric surfactants [29].

The cationic Gemini surfactants adsorb very efficiently on metallic and nonmetallic surfaces and owing to these properties they are used as very effective microbiocides and corrosion inhibitors. Surface, anticorrosion and antimicrobial activity of Gemini surfactants with flexible polymethylene spacers and polymethylene spacers modified by heteroatoms have been extensively studied so far [30,31,32,33,34,35,36,37]. However there are limited data concerning Gemini and oligomeric surfactants with rigid, benzene spacers [38,39,40].

Therefore we have undertaken a systematic study of oligomeric *N*-alkyl-*N*,*N*-dimethylbenzene bromides, i.e., 1,4-bis-[*N*-(1-alkyl)-*N*,*N*-dimethylammoniummethyl]benzene dibromides (**2**–**9**), 1,3,5-tris-[*N*-(1-alkyl)-*N*,*N*-dimethylammoniummethyl]benzene tribromides (**10**–**17**) and 1,2,4,5-tetrakis-[*N*-(1-alkyl)-*N*,*N*-dimethylammoniummethyl]benzene tetrabromides (**18**–**23**) in the scope of synthesis, structure, surface properties and antimicrobial activity.

## 2. Results and Discussion

In this work we report the synthesis, physicochemical properties, antimicrobial activity and spectroscopic analysis (FT-IR, ^1^H-NMR, ^13^C-NMR, 2D-NMR) as well as ESI-MS spectrometry and crystal structure of di- tri- and tetrameric alkylammonium surfactants with aromatic spacer. PM5 semiempirical calculations using the WinMopac 2003 program were also performed [41,42,43].

### 2.1. Synthesis

**Scheme 1:**
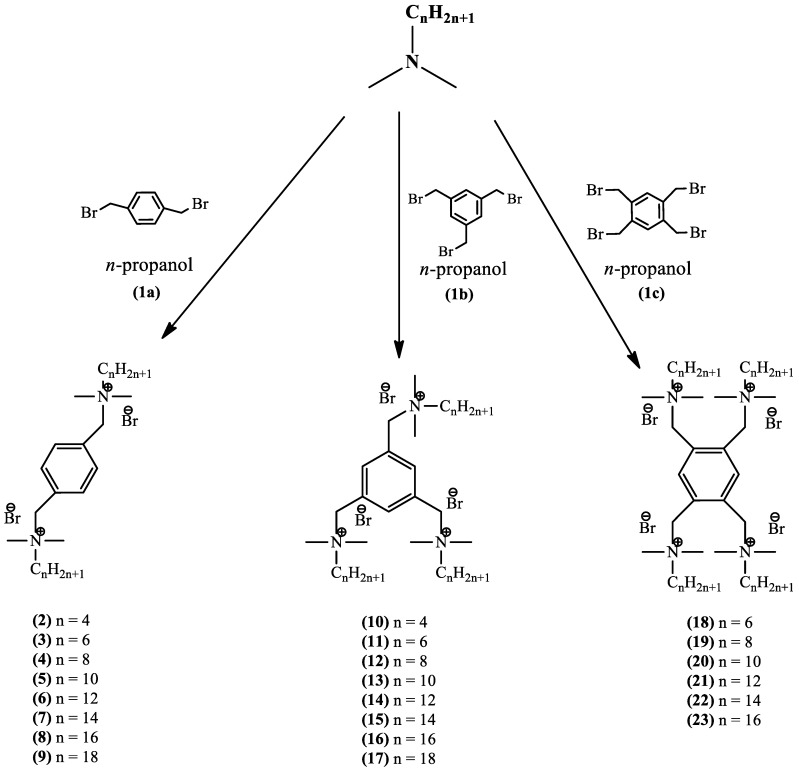
Structures of di- (**2**–**9**) tri- (**10**–**17**) and tetrameric (**18**–**23**) alkylammonium surfactants with aromatic spacer.

Appropriate *N*,*N*-dimethyl-1-alkylamines were quaternized with stoichiometric amounts of bromomethylbenzene derivatives in propanol to give a series of new cationic surfactants: 1,4-bis-[*N*-(1-alkyl)-*N*,*N*-dimethylammoniummethyl]benzene dibromides (**2**–**9**), 1,3,5-tris-[*N*-(1-alkyl)-*N*,*N*-dimethylammoniummethyl]benzene tribromides (**10**–**17**) and 1,2,4,5-tetrakis-[*N*-(1-alkyl)-*N*,*N*-dimethylammoniummethyl]benzene tetrabromides (**18**–**23**) (Scheme 1). Experimental data of synthesized di- tri- and tetrameric alkylammonium surfactants are given in Table S1 (Supplementary material). 1,2,4,5-tetrakis(bromomethyl)benzene (**1c**), which is not commercially available, was prepared by reaction of 1,2,4,5-tetramethylbenzene with *N*-bromosuccinimide according to known literature procedure [44]. In general, reaction time significantly extends with the increase of the degree of oligomerization and the increase of the length of alkyl chain of amine.

**Figure 1:**
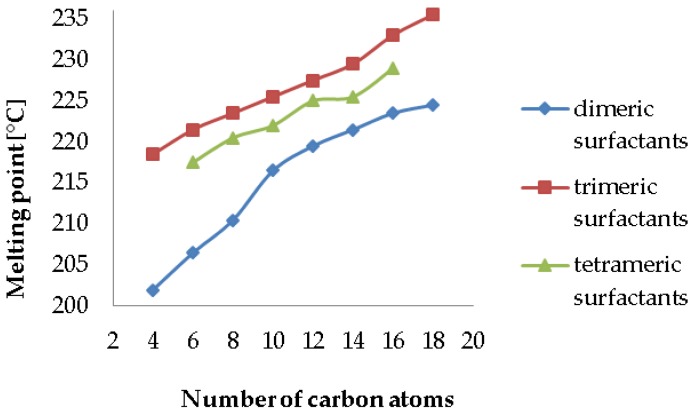
Melting points of dimeric (**2**–**9**), trimeric (**10**–**17**) and tetrameric surfactants (**18**–**23**).

Melting points of the synthesized compounds in each series rise as alkyl chains lengthen . This reflects a better packaging in the crystals (Figure 1).

### 2.2. Spectroscopic Study

#### 2.2.1. ^1^H-NMR, ^13^C-NMR and 2D-NMR Spectra

**Figure 2:**
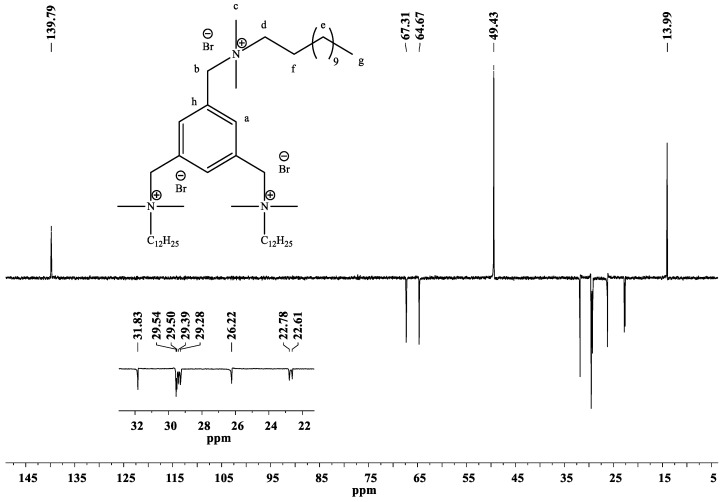
^13^C DEPT spectrum of 1,3,5-tris[(*N*-(1-dodecyl)-*N*,*N*-dimethylammoniummethyl)benzene tribromide (**14**).

^1^H-NMR chemical shifts of compounds **1c**–**23** are given in Tables S2 and S3 (Supplementary material) whereas ^13^C-NMR chemical shifts are given in Tables S4 and S5 (Supplementary material). Representative ^1^H-NMR and ^13^C-NMR spectra are shown in Figures S1 and S2 (Supplementary material). The protons and carbons of terminal methyl groups in alkyl substituents resonate at the lowest values of chemical shifts while those of methylene group attached to positively charged nitrogen atoms are strongly deshielded and lie in the region of the higher values of chemical shifts. To resolve chemical shifts of proton and carbon at methylene groups as well as aromatic carbons the ^13^C DEPT spectrum was generated (compound **14**, Figure 2). It was observed that the signals derived from carbon atoms “a” are shifted towards the highest values, while slightly lower values are assigned to quaternary carbon atoms in benzene “h”.

**Figure 3:**
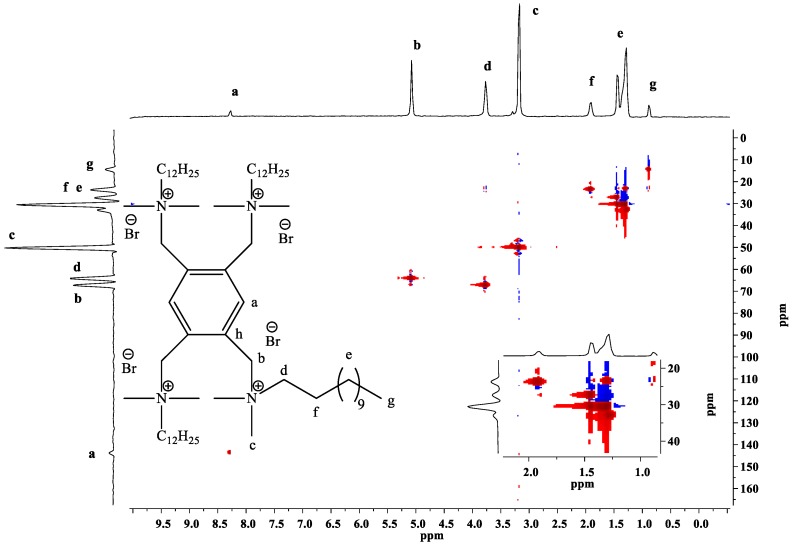
HSQC spectrum (CD_3_OD) of 1,2,4,5-tetrakis[(*N*-(1-dodecyl)-*N*,*N*-dimethylammoniummethyl) benzene tetrabromide (**21**).

**Figure 4:**
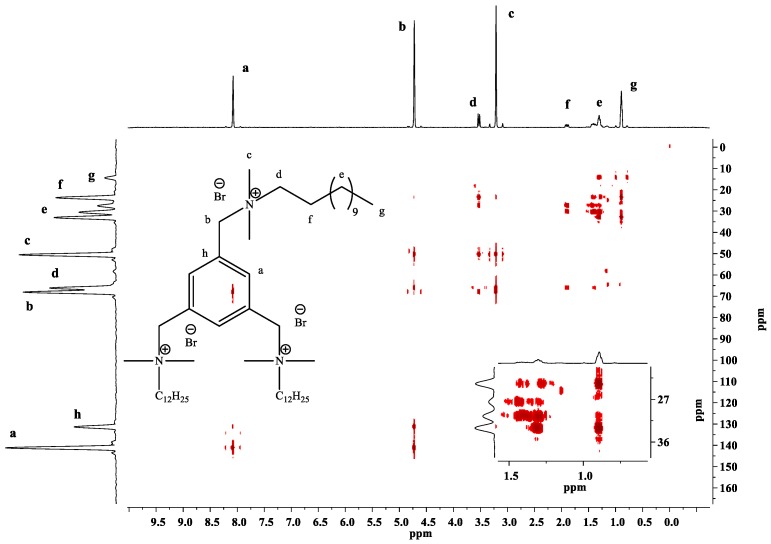
HMBC spectrum (CD_3_OD) of 1,3,5-tris[(*N*-(1-dodecyl)-*N*,*N*-dimethylammoniummethyl)benzene tribromide (**14**).

To get more accurate information about chemical shifts, a correlation spectra COSY and HSQC were measured. The conjunction of the HSQC and COSY allows correlation of the bonds between the proton originating from one carbon atom and signals from nuclei of the same element, spaced by not more than three bonds, respectively. Careful analysis has confirmed the structure of the compounds and revealed a direct correlation between the atoms of hydrogen-hydrogen and carbon-hydrogen bonds. Moreover, HMBC technique allowed correlation of the signals from the nuclei of various elements spaced by 3 or 5 bonds (Figure S3, Supplementary material). Figure 3 shows HSQC spectrum for compound **21** and Figure 4 shows HMBC spectrum for compound **14**.

#### 2.2.2. FT-IR Spectra

**Figure 5:**
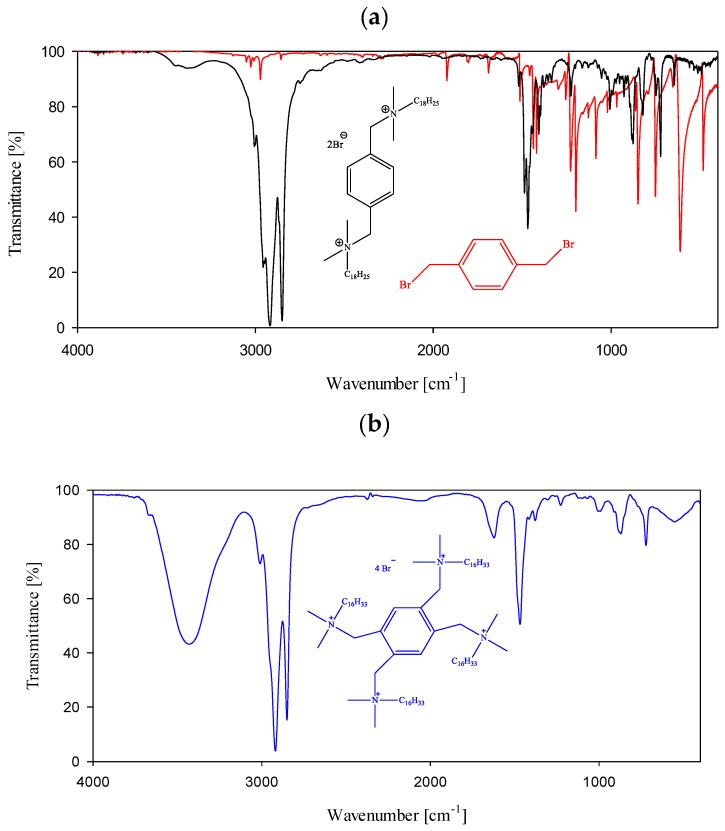
FT-IR spectra (KBr pellets) of 1,2-di(bromomethyl)benzene (―) and **9** (**―**) (**a**) and **23** (―) (**b**).

FT-IR spectra of 1,2-di(bromomethyl)benzene and compound **9** and **23** are shown in Figure 5. The characteristic features of the FT-IR spectra for dimeric (**2**–**9**), trimeric (**10**–**17**) and tetrameric (**18**–**23**) quaternary ammonium salts are a broad intense absorption of stretching asymmetric (ν_as_) and symmetric (ν_s_) vibrations of methyl and methylene groups at 3000–2840 cm^−1^ and deformation bands, asymmetric and symmetric vibrations (δ_s_, δ_as_) of –CH_3_ group at 1470–1430 cm^−1^ and 1395–1365 cm^−1^ as well as deformation vibrations (δ) of –CH_2_ group at 1475–1450 cm^−1^. Moreover, absorption bands of benzene ring, i.e., stretching vibrations of the (=C–H) group (ν) at 3050–3010 cm^−1^, skeletal vibrations of the C–C group at 1620–1430 cm^−1^ (four bands), in-plane deformation vibrations (δ_ip_) at 1250–950 cm^−1^ and out-of-plane deformation vibrations (δ_oop_) at 900-650 cm^−1^ (of =C–H group) are also observed. The disappearance of the stretching vibrations band of C-Br group at 700–500 cm^−1^ upon quaternization confirm the formation of alkylammonium products (Figure 5a). The strong absorption at the 3450–3350 cm^−1^ region in (**23**) arises from the OH stretching vibrations (Figure 5b). 

#### 2.2.3. PM5 Calculations

**Figure 6:**
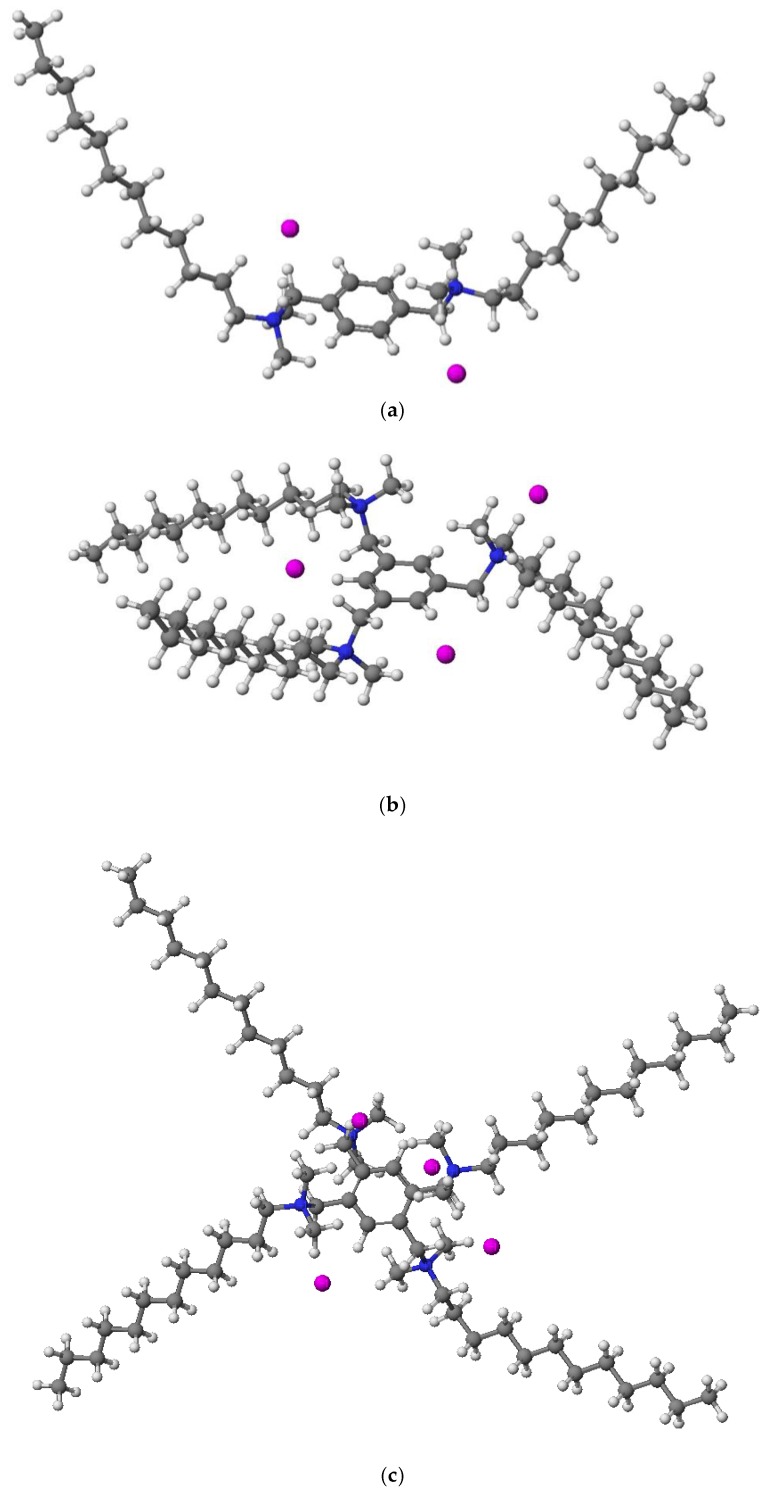
Molecular models of representative compounds **6** (**a**), **14** (**b**) and **21** (**c**) calculated by the PM5 method.

PM5 semiempirical calculations were performed using the WinMopac 2003 program. Representative compounds (**6**, **14**, **21**) are shown in Figure 6. The final heats of formation (HOF) for all dimeric (**2**–**9**), trimeric (**10**–**17**) and tetrameric (**18**–**23**) quaternary ammonium salts are presented in Table 1.

**Figure 7:**
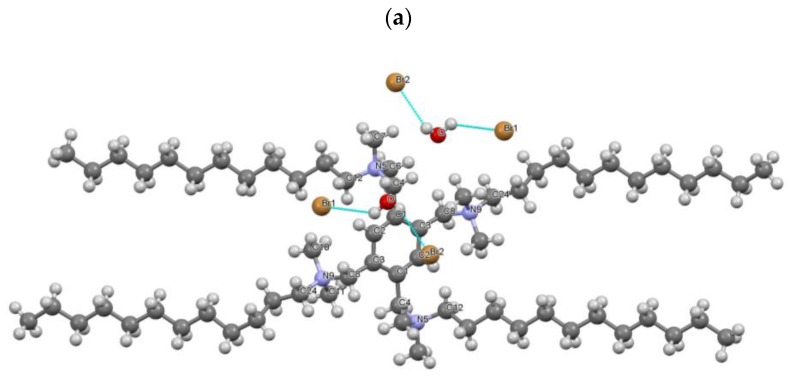

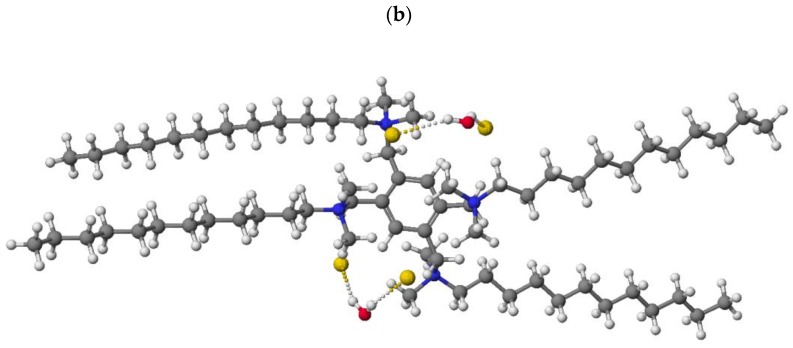
Comparison of the X-ray (**a**) and PM5 (**b**) structures of compound **21**.

The lowest values of HOF from among all quaternary ammonium salts (**2**–**23**) are observed for tetrameric (**18**–**23**) substituted derivatives. This is consistent with the results obtained for 1,4-bis(bromomethyl)benzene (**1a**), 1,3,5-tris(bromomethyl)benzene (**1b**) as well as 1,2,4,5-tetrakis(bromomethyl)benzene (**1c**), wherein HOF is decreasing from disubstituted to tetrasubstituted benzene derivative (Table 1). In general the HOF of 1,2,4,5-tetrasubstituted benzene derivatives were lower than those of 1,3,5-tri- and 1,4-disubstituted benzene derivatives. A similar relationship was observed in our previous work [45,46]. This is related to symmetry and the convertible position of long hydrocarbon chains of molecules. Simultaneously, an increase of the length of an alkyl chain also reduces HOF. These compounds are stabilized by electrostatic interactions that arise from the quaternary nitrogen atoms as well as bromide anion. In addition, in the semiempirical calculations we included the London Dispersion Forces between the hydrocarbon chains, which is illustrated by the increase in melting compounds. Furthermore, the presence of two water molecules stabilizes the molecule (**21**) and reduces the HOF to −328.4818 kcal/mol (Figure 7b). This value is nearly 100 kcal/mol lower than the isolated molecule (see Table 1).

#### 2.2.4. X-ray Analysis

**Figure 8:**
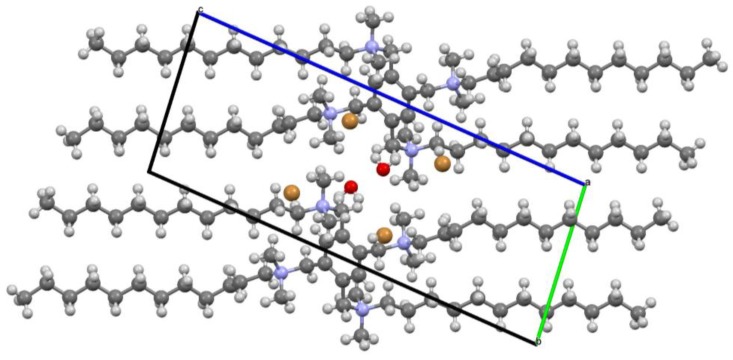
Crystal packing of compound **21**.

1,2,4,5-Tetrakis-[*N*-(1-dodecyl)-*N*,*N*-dimethylammoniummethyl)benzene tetrabromide crystallizes in the triclinic space group P-1. Molecular aggregation of compound (**21**) is governed by ionic interactions between bromine anions and positively charged nitrogen atoms, and by attractive H-H interactions between C_12_H_25_ hydrocarbon chains [47]. The water molecule is hydrogen-bonded to the bromine anions. The composition of compound (**21**) molecules is reflected in the molecular packing. The crystal of tetrameric quaternary ammonium salts with benzene ring (**21**) in spacer is built of hydrophilic sub-layers containing the bromine anions and nitrogen cations, and of hydrophobic sub-layers consisting of –C_12_H_25_ hydrocarbon chains. This results in a structure with alternating hydrophilic and hydrophobic layers, in which the aggregation is governed by distinct forces. In the hydrophilic layer, the ionic interaction between bromine and nitrogen is accompanied by eight CH···Br and two OH···Br hydrogen bonds. Selected details for compound (**21**) about the crystal structure, experiment and structure solution and refinement are given in Table 2. Figure 7a and Figure 8 shows molecular structure and crystal packing of compound (**21**) respectively. Summary of X-ray data for (**21**) are given in CCDC 1554259.

#### 2.2.5. Aggregation Behavior

**Figure 9:**
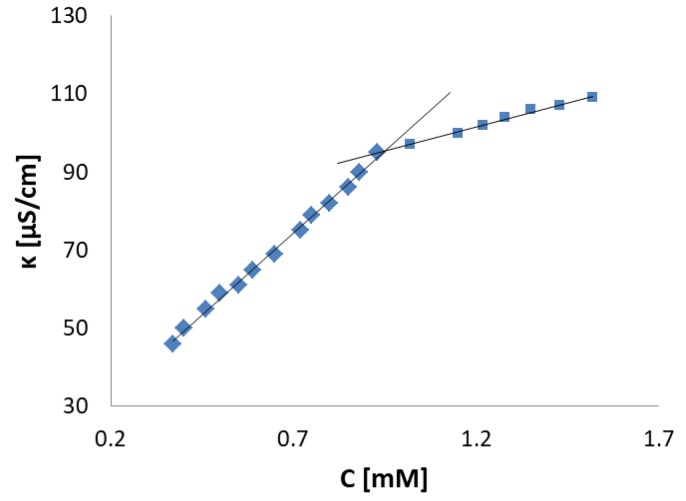
Specific conductivity (κ) versus concentration of **7** in water at 50 °C.

The variation of the specific conductivity in aqueous solutions of the compounds (**6**–**9**) as a function of the concentration was investigated. Conductivity measurements were carried out at 50 °C due to low solubility of compounds in water. Representative conductometric profile of surfactant (**7**) is displayed in Figure 9.

The CMC values of dimeric surfactants decrease with increasing number of carbon atoms in the hydrophobic chain (Table 3). There was observed no clear dependence between the β parameter (counterion binding parameter) value and the length of hydrophobic chain in the molecule (Table 3). It was calculated that compounds with dodecyl, tetradecyl and hexadecyl chain have the highest values, which may be caused by a stronger binding counterion by aggregate molecules compared to compounds with short alkyl chains. The average Gibbs energy takes negative values for all of the surfactants what indicates that the aggregation process is spontaneous. The value of ΔG°_mic_ for compounds with longer alkyl (C12, C14 and C16) are much smaller what clearly show that these compounds are less agile than the free ions in solution. 

#### 2.2.6. Antimicrobial Properties

Minimal inhibitory concentrations (MIC), i.e., minimum concentration of the preparation at which further growth of tested microorganisms is stopped, of 1,4-bis-[*N*-(1-alkyl)-*N*,*N*-dimethylammonium-methyl]benzene dibromides have been determined against bacteria, *S.aureus*, *B. subtilis*, *E. coli* and *P. aeruginosa* as well as microscopic fungi, *C. albicans*, *A. niger* and *P. chrysogenum* (Table 4).

**Figure 10:**
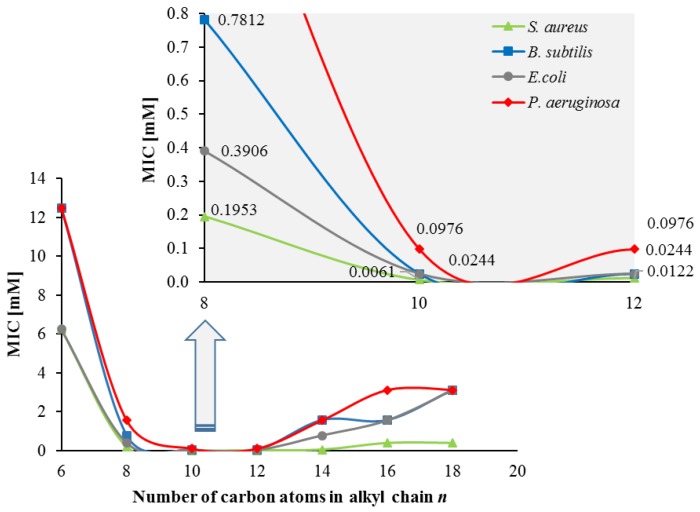
Antibacterial activity of compounds **2**–**9**.

The MIC values of 1,4-bis-[*N*-(1-alkyl)-*N*,*N*-dimethylammoniummethyl]benzene dibromides for bacteria range from 0.0122 to 12.5 mM. The shorter hydrocarbon chain is not able to penetrate the cell wall and their minimal concentration, which inhibits growth of bacteria, is above 6 mM. The highest antibacterial activity are observed for derivatives that have 10–12 carbon atoms in the alkyl substituent (Figure 10). The elongation of the hydrocarbon chain to 14–18 methylene group causes increases the MIC values. The least susceptible bacteria to chemical substances is *Pseudomonas aeruginosa*. These Gram-negative rots very often cause the formation of biofilms on many surfaces in cosmetic and food industry and also in medical environment.

**Figure 11:**
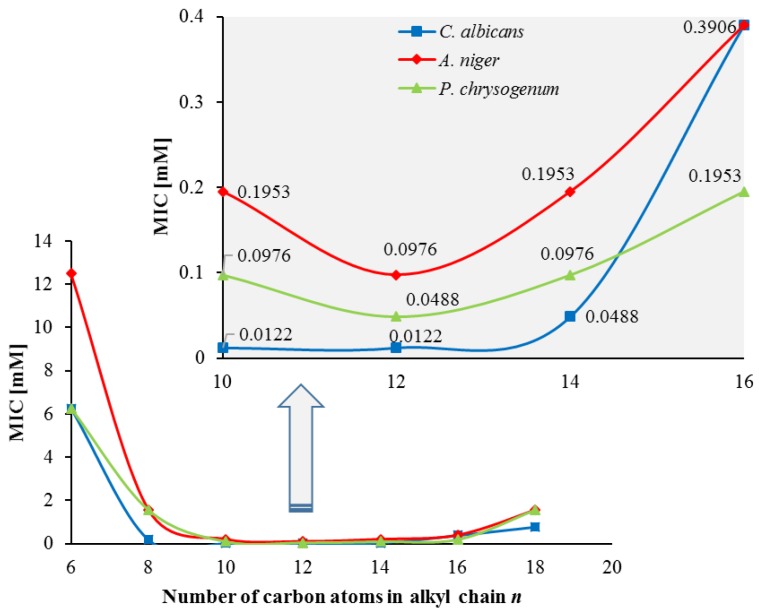
Antifungal activity of compounds **2**–**9**.

The range of MIC values of 1,4-bis-[*N*-(1-alkyl)-*N*,*N*-dimethylammonium-methyl]benzene dibromides, analogues of benzalkonium chlorides (BAC), for microscopic fungi *C. albicans*, *A. niger* and *P. chrysogenum* are the same like for bacteria 0.0122–12.5 mM (Table 4). However the lowest concentrations, above 0.2 mM, have compounds that contain 10–14 carbon atoms in the alkyl substituent (Figure 11). The highest MIC values are obtained for the derivatives with the shortest C4–C8 alkyl chains. Among the three tested strains of microscopic fungi the least sensitive to the effects of derivative BAC was *A. niger*.

Comparing the MIC values (Table 4) of all tested microorganisms, it was observed that the lowest concentrations were obtained for 1,4-bis-[*N*-(1-dodecyl)-*N*,*N*-dimethylammoniummethyl]benzene dibromide (**6**). Quaternary ammonium salts containing at least one chain of 12 carbon atoms are also most commonly described in the literature. Among them is benzalkonium chloride (BAC), which is often used in many industries and medicine as a component of biocidal preparations [48,49,50]. Received by Fazlara et al. and Merianos MIC values for BAC are generally higher compared to 1,4-bis-[*N*-(1-alkyl)-*N*,*N*-dimethylammoniummethyl]benzene dibromides presented at work (Table 5) [49,51]. Exceptions are the results obtained by El Hage et al., which obtained lower benzalkonium chloride concentrations for *S. aureus* and partially *A. niger* [52]. The same species of bacteria or fungi, but different strains may have different sensitivities for the same compound as Koziróg and Brycki write in their work [53]. Thus, compounds containing decyl carbon chains for MIC values for this strain may be ordered in ascending order as 1,4-bis-[*N*-(1-decyl)-*N*,*N*- dimethylammoniummethyl]benzene dibromide (**5**) < BAC < Benzyl-*N*-decyl-dimethylammonium bromide < *N*,*N*-dimethyl-*N*-(4-methylpyridyl)-*N*-decyl-ammonium chlorides. However, the MIC results for compounds containing from 12 to 16 methylene groups in the chain are higher in comparison with those obtained by El Hage et al., but lower than for pyridyl analogues of BAC [52,54].

## 3. Materials and Methods

### 3.1. General Information

All chemicals were commercial products from Sigma-Aldrich (Poznań, Poland). Infrared spectra of products were recorded in the KBr (in the frequency range 4000–400 cm^−1^) pellets in temperature 298 K using Bruker FT-IR IFS 66/s spectrometer (Karlsruhe, Germany), evacuated to avoid water and CO_2_ absorptions, at 2 cm^−1^ resolution. The ^1^H-NMR and ^13^C-NMR spectra were measured Varian Mercury 300/400 MHz spectrometer (Oxford, UK) in CDCl_3_ or CD_3_OD, and TMS was used as an internal standard. 2D-NMR spectra of products were recorded using spectrometer Bruker Avance 600MHz spectrometer (Billerica, MA, USA) operating at the frequencies 600.281 MHz and 150.947 MHz for ^1^H and ^13^C respectively, and equipped with a 5 mm triple-resonance inverse probe head [^1^H/^31^P/BB] with a self-shielded *z* gradient coil (90° ^1^H pulse width 9.0 μs and ^13^C pulse width 13.3 μs) with CDCl_3_ as solutions. The ESI (electron spray ionization) mass spectra were recorded on a Waters/Micromass (Manchester, UK) ZQ mass spectrometer equipped with a Harvard Apparatus (Saint Laurent, QC, Canada), syringe pump. The standard ESI-MS mass spectra were recorded at the cone voltage 30 V. Melting point was determined using Stuart SMP10 apparatus. Single-crystal diffractometer Xcalibur EOS CCD with Mo Kα X-ray source was used for diffraction measurements. The sample solutions were prepared in methanol at the concentration of approximately 10^−5^ M. Determination of the UB-matrices and initial data reduction were performed using the CrysAlisPro program suite [54]. CCDC 1554260 contains the supplementary crystallographic data for this paper. These data can be obtained free of charge via http://www.ccdc.cam.ac.uk/conts/retrieving.html (or from the CCDC, 12 Union Road, Cambridge CB2 1EZ, UK; Fax: +44 1223 336033; E-mail: deposit@ccdc.cam.ac.uk). All structures were solved by direct methods with SHELXS and refined with anisotropic displacement parameters for non-H atoms by program SHELXL-97, using the OLEX2 interface [55,56]. Hydrogen atoms were located in the ideal position from molecular geometry and refined according the “riding” model.

### 3.2. Synthesis

1,2,4,5-tetrakis(bromomethyl)benzene (**1c**) was obtained by the reaction of 1,2,4,5-tetramethylbenzene (2.8 g; 20 mmol) with NBS (14.4 g; 80 mmol) and benzoyl peroxide (1 g; 5.9 mmol) in tetrachloromethane (50 mL). Reagents were heated at 77 °C for 50 min. The precipitate was filtered off under reduced pressure and washed with hot tetrachloromethane. The filtrate was concentrated to small volume and cooled to 4 °C. Crude product was filtrated and purified by crystallization from methanol. Rt< 1 h, white solid (15%), m.p. 159–160 °C. ^1^H-NMR (CDCl_3_) δ: 7.37 ppm (2H, a), 4.60 ppm (8H, b). ^13^C-NMR (CDCl_3_) δ: 137.6 ppm (a), 133.6 ppm (h), 28.7 ppm (b). FT-IR (KBr) ν_max_: 3026, 2980, 2933, 1504, 1456, 1210, 911, 797, 678, 598.

#### Series of dimeric quaternary ammonium salts (**2**–**9**). 

1,4-bis-[*N*-(1-alkyl)-*N*,*N*-dimethylammoniummethyl]benzene dibromides (**2**–**9**) were synthesized of 1 equivalent of 1,4-di(bromomethyl)benzene (0.5 g; 1.89 mmol) (**1a**) with 2 equivalents (3.78 mmol) of *N*-butyl-*N*,*N*-dimethylamine (0.38 g), *N*-hexyl-*N*,*N*-dimethylamine (0.49 g), *N*,*N*-dimethyl-*N*-octylamine (0.59 g), *N*-decyl-*N*,*N*-dimethylamine (0.7 g), *N*-dodecyl -*N*,*N*-dimethylamine (0.81 g), *N*,*N*-dimethyl-*N*-tetradecylamine (0.91 g), *N*-hexadecyl -*N*,*N*-dimethylamine (1.02 g), *N*,*N*-dimethyl-*N*-octadecylamine (1.13 g), respectively, by heating in *n*-propanol from 4 h to 10 h. White solids were obtained. The crude products were purified by recrystallization from a mixture of acetone/methanol (10:1) (Supplementary material).

#### Series of trimeric quaternary ammonium salts (**10**–**17**). 

1,3,5-tris-[*N*-(1-alkyl)-*N*,*N*-dimethylammoniummethyl]benzene tribromides (**10**–**17**) were synthesized of 1 equivalent of 1,3,5-tri(bromomethyl)benzene (0.5 g; 1.4 mmol) (**1b**) with 3 equivalents (4.2 mmol) of *N*-butyl-*N*,*N*-dimethylamine (0.43 g), *N*-hexyl-*N*,*N*-dimethylamine (0.54 g), *N*,*N*-dimethyl-*N*-octylamine (0.66 g), *N*-decyl-*N*,*N*-dimethylamine (0.77 g), *N*-dodecyl-*N*,*N*-dimethylamine (0.9 g), *N*,*N*-dimethyl-*N*-tetradecylamine (1.03 g), *N*-hexadecyl-*N*,*N*-dimethylamine (1.13 g), *N*,*N*-dimethyl-*N*-octadecylamine (1.25 g), respectively, by heating in *n*-propanol from 4 h to 15 h. White solids were obtained. The crude products were purified by recrystallization from a mixture of acetone/methanol (10:1) (Supplementary material).

#### Series of tetrameric quaternary ammonium salts (**18**–**23**). 

1,2,4,5-tetrakis-[*N*-(1-alkyl)-*N*,*N*-dimethylammoniummethyl]benzene tetrabromides (**18**–**23**) were synthesized of 1 equivalent of 1,2,4,5-tetra(bromomethyl)benzene (0.5 g; 1.12 mmol) (**1c**) with 4 equivalents (4.48 mmol) of *N*-hexyl-*N*,*N*-dimethylamine (0.58 g), *N*,*N*-dimethyl-*N*-octylamine (0.7 g), *N*-decyl-*N*,*N*-dimethylamine (0.83 g), *N*-dodecyl-*N*,*N*-dimethylamine (0.95 g), *N*,*N*-dimethyl-*N*-tetradecylamine (1.08 g), *N*-hexadecyl-*N*,*N*-dimethylamine (1.23 g), respectively, by heating in *n*-propanol from 7 h to 14 h. White solids were obtained. The crude products were purified by recrystallization from a mixture of acetone/methanol (10:1) (Supplementary material).

### 3.3. Critical Micellization Concentration

Measurements of conductivity were obtaining using a CO300 conductivity meter (VWR, Gdansk, Poland) with graphite electrode in conjunction with a thermostat. CMC values of compounds **3**–**9** and **14** were obtained from conductometric titrations conducted in double distilled water in 50 °C.

### 3.4. Antimicrobial Properties

The MIC values of 1,4-bis-[*N*-(1-alkyl)-*N*,*N*-dimethylammoniummethyl]benzene dibromides (from **4** to **18**) against bacteria and microscopic fungi (yeast and molds) were measured at the Institute of Fermentation Technology and Microbiology, Technical University of Lodz in Poland. The MICs are defined as the lowest concentration of the compounds at which there was no visible growth of microorganisms. MIC values were determined by a standard tube 2-fold dilution method [29]. The experiments were carried out on the Gram-positive bacteria: *Staphylococcus aureus* ATCC 6538, *Bacillus subtilis* NCAM01644, Gram-negative bacteria: *Escherichia coli* ATCC 10536, *Pseudomonas aeruginosa* ATCC 85327, yeast: *Candida albicans* ATCC 10231, and molds: *Aspergillus niger* ATCC 16404, *Penicillium chrysogenum* ATCC 60739. Antifungal properties were checked on a Malt Extract Broth medium (Merck, Darmstadt, Germany) at a density of inoculum of 1–2 × 10^6^ cfu/mL; antibacterial activity—on Trypticase Soy Broth (Merck), density of inoculum 1–2 × 10^7^ cfu/mL. All tests were repeated three times.

## 4. Conclusions

Three series of dimeric, trimeric and tetrameric quaternary alkylammonium salts with benzene spacers were synthesized by S_N_2 reaction of bromomethylbenzene derivatives with tertiary alkyldimethylamines with good yield. The structure and purity of new surfactants were confirmed by FT-IR, ^1^H-NMR, ^13^C-NMR and 2D-NMR, mass spectroscopy (ESI-MS) and elemental analysis. The structure of one dimeric surfactant (**9**) was confirmed by X-ray analysis. The PM5 semi-empirical method proved that tetrameric surfactants are more stable than trimeric and dimeric ones. Moreover, it has been shown that molecules of water additionally stabilize the structure of the surfactant. Aggregation behavior of the synthesized compounds depends on the substituent, and CMC values decrease as alkyl chain lengthens. Dimeric surfactants, which are soluble in water, exhibit strong antimicrobial activity against bacteria (*Staphylococcus aureus*, *Bacillus subtilis*, *Escherichia coli*, *Pseudomonas aeruginosa*) and fungi (*Aspergillus niger*, *Candida albicans*, *Penicillium chrysogenum*). The biocidal efficacy depends on the length of alkyl chain and is the strongest for decyl and dodecyl derivatives. 

## Figures and Tables

**Table 1:** Heat of formation (HOF) [kcal/mol] of di-, tri- and tetrabromosubstituted derivatives of benzene (**1a**, **1b**, **1c**) and its quaternary ammonium salts (**2**–**23**).

Compound	Heat of Formation [kcal/mol]	ΔHOF [kcal/mol]
**1a**	10.6060	–
**1b**	5.6268	–
**1c**	3.0680	–
**2**	−2.1145	8.4915
**3**	−24.5953	−13.9893
**4**	−46.7459	−36.1399
**5**	−68.8809	−58.2749
**6**	−91.0872	−80.4812
**7**	−113.3524	−102.7464
**8**	−135.6207	−125.0147
**9**	−158.0240	−147.4180
**10**	−34.9103	−5.6268
**11**	−68.3429	−62.7161
**12**	−101.8142	−107.4410
**30**	−135.2579	−140.8847
**14**	−155.2349	−160.8617
**15**	−213.0509	−218.6777
**16**	−233.2146	−238.8414
**17**	−266.9034	−272.5302
**18**	−94.7088	−97.7768
**19**	−130.8176	−133.8856
**20**	−184.1519	−187.2199
**21**	−228.7916	−231.8596
**22**	−280,4156	−283,4836
**23**	−325,1616	−328,2296

ΔHOF = HOF_compounds_ (**2**–**23**) − HOF_bromobenzene_.

**Table 2:** Crystal data and structure refinement for compound **21**.

Empirical Formula	C_66_H_138_Br_4_N_4_O_2_
Formula weight	1339,44
Temperature	293(2) K
Wavelength	0.71073 Å
Crystal system, space group	Triclinic, P-1
Unit cell dimensions	a: 8.6269(17) b: 9.2619(13) c: 23.787(4) α: 96.864(13) β: 99.006(15) γ: 90.157(14)
Volume	1863.3(6) Å^3^
Z; Dx	2; 1194 mg/mm^3^
µ	2200 mm^−1^
F(000)	718
θ Range for data collection	6.354–53.284
Limiting indices	−10 ≤ h ≤ 10, −11 ≤ k ≤ 11, −29 ≤ l ≤ 29
Reflections collected/unique	27738/7298 (0,0111) 0.927
R(F) I > 2σ (I)	0.1306
wR(F^2^)	0.1437
max/min Δρ	0.45/−0.40 e/Ǻ^−3^

**Table 3:** Critical micelle concentration (CMC), counterion binding parameter (β) and Gibbs free energy of micellization (ΔG°_mic_) of compounds (**3**–**9**) obtained by conductivity measurements.

Compound	CMC [mM]	ΔG°_mic_ [kJ/mol]	β
**3**	12.38	−18.12	0.19
**4**	11.67	−19.15	0.22
**5**	7.27	−19.25	0.16
**6**	1.2	−39.01	0.53
**7**	0.92	−43.88	0.62
**8**	0.48	−38.81	0.40
**9**	0.29	−28.79	0.15

**Table 4:** Minimal inhibitory concentrations (MIC [mM]) of 1,4-bis-[*N*-(1-alkyl)-*N*,*N*-dimethylammonium-methyl]benzene dibromides.

Compound	*S. aureus*	*B. subtilis*	*E. coli*	*P. aeruginosa*	*C. albicans*	*A. niger*	*P. chrysogenum*
**2**	>12.5	>12.5	>12.5	>12.5	>12.5	>12.5	>12.5
**3**	6.2500	12.5000	6.2500	12.5000	6.2500	12.5000	6.2500
**4**	0.1953	0.7812	0.3906	1.5625	0.1953	1.5625	1.5625
**5**	0.0061	0.0244	0.0244	0.0976	0.0122	0.1953	0.0976
**6**	0.0122	0.0244	0.0244	0.0976	0.0122	0.0976	0.0488
**7**	0.0488	1.5625	0.7812	1.5625	0.0488	0.1953	0.0976
**8**	0.3906	1.5625	1.5625	3.1250	0.3906	0.3906	0.1953
**9**	0.3906	6.2500	6.2500	3.1250	0.3906	0.7812	0.7812

**Table 5:** MIC values for benzalkonium chloride derivatives containing 10–16 carbon atoms in aliphatic chains—literature data [49,50,51,52,54].

Chemical Substance	Alkyl Chain	Microorganisms			Literature
		*S. aureus*	*P. aeruginosa*	*A. niger*	
BAC	10	0.0048 **	1.25 *	0.625 *	
	12	0.0024 **	0.312 *	0.0781 *	El Hage et al. [52]
	14	0.0006 **	0.0781 *	0.0195 *	
	16	0.0006 **	0.156 *	0.156 *	
	10	1.44 *	3.85	nt	
	12	0.13 *	0.35		Merianos [51]
	14	0.04 *	0.11		
	16	0.076 *	0.5		
	12	0.122 *	0.183	nt	Fazlara et al. [49]
	12	0.0028	0.175	nt	Feder-Kubis et al. [50]
*N*,*N*-dimethyl-*N*-(4-methylpyridyl)-*N*-alkyl-ammonium chlorides	10	1.5625 *	1.5625	12.499 *	
	12	0.1953 *	0.7812	1.5625 *	
	14	0.0488 *	0.1953	0.3906 *	Brycki et al. [54]
	16	0.0244 *	0.0976	0.1953 *	
Benzyl-*N*-alkyl-dimethyl-ammonium bromide	10	0.0195 **	1.25 *	0.625 *	
	12	0.0048 **	0.156 *	0.0781 *	El Hage et al. [52]
	14	0.0024 **	0.078 *	0.0195 *	
	16	0.0012 **	0.312 *	0.0781 *	

(* or **) the same strains of the species; BAC benzyl-*N*-alkyl-dimethylammonium chloride.

## Supplementary Materials

Supplementary materials are available online. **Figure S1:** 2D NMR spectra of compound **21**: COSY (a), HSQC (b) and HMBC (c), **Table S1:** Experimental data of synthesized di- tri- and tetrameric alkylammonium surfactants with aromatic spacer and with different chain lengths, **Table S2:** The ^1^H NMR chemical shifts (ppm) of compounds **1c-10**, **Table S3:** The ^1^H NMR chemical shifts (ppm) of compounds **11**–**23**, **Table S4:** The ^13^C NMR chemical shifts (ppm) of compounds **1c-10**, **Table S5:** The ^13^C NMR chemical shifts (ppm) of compounds **11**–**23**.
