# Supplementary material for: Synthesis, Structure, Surface and Antimicrobial Properties of New Oligomeric Quaternary Ammonium Salts with Aromatic Spacers
Source: Molecules. 2017 Oct 25;22(11):1810. doi: 10.3390/molecules22111810 (PMC6150277; doi:10.3390/molecules22111810)
Supplement: Supplementary file 1 [file molecules-22-01810-s001.pdf]

Synthesis, Structure, Surface and Antimicrobial Properties of New Oligomeric Quaternary Ammonium Salts with Aromatic Spacer

Bogumił Brycki[1,*], Anna Koziróg[2], Iwona Kowalczyk[1], Tomasz Pospieszny[1], Paulina Materna[1], Jędrzej Marciniak[3]

[1] Laboratory of Microbiocides Chemistry, Faculty of Chemistry, Adam Mickiewicz University in Poznań, Umultowska 89b, 61-614 Poznań, Poland, E-Mails: brycki@amu.edu.pl (B.B.); iwkow@amu.edu.pl (I.K.); tposp@amu.edu.pl (T.P.); p_materna@wp.pl (P.M.);

[2] Institute of Fermentation Technology and Microbiology, Faculty of Biotechnology and Food Sciences, Lodz University of Technology, Wólczańska 171/173, 90-924 Łódź, Poland; E-Mails: anna.kozirog@p.lodz.pl (A.K.)

[3] Department of Materials Chemistry, Faculty of Chemistry, Adam Mickiewicz University in Poznań, Umultowska 89b, 61-614 Poznań, Poland, E-Mail: jedrzej.marciniak@amu.edu.pl (J.M.).

* Authors to whom correspondence should be addressed; E-Mails: brycki@amu.edu.pl (B.B.); phone: +48-61-829-1694 (B.B.).

Table S1. Experimental data of synthesized di- tri- and tetrameric alkylammonium surfactants with aromatic spacer and with different chain lengths.

Compound	Molar mass [g/mol]	Reaction Time [h]	Yield [%]	Melting point [°C]	Molecular formula	% N		% C		% H	
						AE	calc.	AE	calc.	AE	calc.
2	466,35	4	90	201-203	$C_{20}H_{38}N_2Br_2$	5,93	5,93	50,43	50,96	8,44	9,39
3	522,45	6	80	206-207	$C_{24}H_{46}N_2Br_2$ x 0,5 H_2O	5,34	5,27	54,19	54,24	9,12	8,91
4	578,56	8	90	210-211	$C_{28}H_{54}N_2Br_2$	4,99	4,84	58,26	58,13	9,60	9,41
5	634,67	8	90	216-217	$C_{32}H_{62}N_2Br_2$	4,54	4,41	60,33	60,56	10,03	9,85
6	690,78	9	95	219-220	$C_{36}H_{70}N_2Br_2$	4,28	4,06	62,34	62,61	10,39	10,21
7	746,89	9	95	221-222	$C_{40}H_{78}N_2Br_2$	3,66	3,75	64,22	64,33	10,69	10,53
8	802,99	10	90	223-224	$C_{44}H_{86}N_2Br_2$	3,56	3,49	65,76	65,81	10,98	10,79
9	859,1	10	90	224-225	$C_{48}H_{94}N_2Br_2$	3,40	3,26	67,43	67,11	11,26	11,03
10	660,46	4	50	218-219	$C_{27}H_{54}N_3Br_3$ x H_2O	5,76	6,19	47,33	47,80	8,53	8,32
11	744,62	6	50	221-222	$C_{33}H_{66}N_3Br_3$ x 1,5 H_2O	5,25	5,45	51,64	51,37	9,22	9,01

12	828,79	7	60	223-224	$C_{39}H_{78}N_3Br_3$ x H_2O	4,69	4,96	55,41	55,32	9,78	9,52
13	912,95	8	40	225-226	$C_{45}H_{90}N_3Br_3$ x H_2O	4,28	4,51	58,39	58,06	10,14	9,96
14	997,11	10	70	227-228	$C_{51}H_{102}N_3Br_3$ x 2 H_2O	4,03	4,07	59,06	59,29	10,38	10,34
15	1081,27	12	60	229-230	$C_{57}H_{114}N_3Br_3$ x 2 H_2O	3,63	3,76	61,01	61,28	10,59	10,64
16	1165,43	13	50	232-234	$C_{63}H_{126}N_3Br_3$ x 1,5 H_2O	3,34	3,52	64,20	63,46	11,10	10,90
17	1249,6	15	85	235-236	$C_{69}H_{138}N_3Br_3$ x H_2O	3,23	3,31	65,45	65,38	11,29	11,13
18	966,79	7	15	217-219	$C_{42}H_{86}N_4Br_4$ x H_2O	5,51	5,69	51,36	51,22	9,34	9,01
19	1079,01	9	5	220-221	$C_{50}H_{102}N_4Br_4$ x 2 H_2O	4,69	5,02	54,69	53,86	9,83	9,58
20	1191,22	10	30	221-223	$C_{58}H_{118}N_4Br_4$ x H_2O	4,47	4,63	57,34	57,61	10,05	10,00
21	1303,44	12	70	224-226	$C_{66}H_{134}N_4Br_4$ x 2 H_2O	4,24	4,18	59,22	59,18	10,52	10,38
22	1415,63	13	50	225-226	$C_{74}H_{150}N_4Br_4$ x H_2O	4,13	3,91	61,65	62,00	10,62	10,69
23	1527,84	14	62	228-230	$C_{82}H_{166}N_4Br_4$ x H_2O	3,73	3,62	63,46	63,71	10,73	10,95

Table S2. The ^1H NMR chemical shifts (ppm) of compounds **1c-10**.

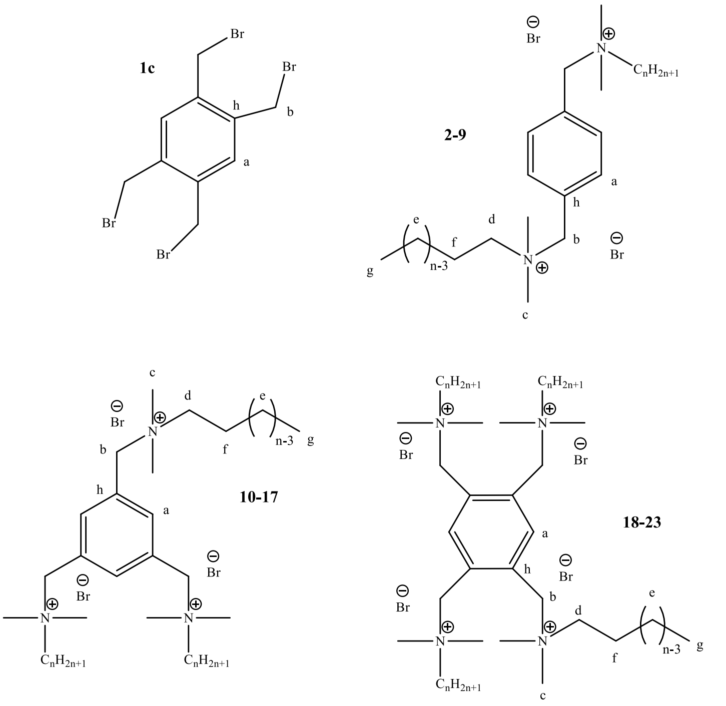

The structures and numbering protons and carbons of compounds **1c-23**.

Protons	1c[b]	2[a]	3[a]	3[b]	4[a]	4[b]	5[a]	5[b]	6[a]	6[b]	7[a]	8[a]	9[a]	10[a]	10[b]
a	7.37	7.76	7.76	7.86	7.75	7.84	7.75	7.84	7.75	7.82	7.75	7.75	7.74	8.09	8.01
b	4.60	4.66	4.66	5.28	4.64	5.29	4.65	5.29	4.64	5.28	4.64	4.64	4.64	4.73	4.83
c	-	3.11	3.11	3.27	3.10	3.26	3.10	3.26	3.10	3.25	3.10	3.09	3.10	3.22	3.11
d	-	3.43	3.41	3.61	3.40	3.60	3.40	3.60	3.39	3.59	3.40	3.38	3.38	3.54	3.42
e	-	1.46	1.41	1.37	1.33, 1.41	1.37, 1.27	1.30, 1.41	1.37, 1.26	1.28, 1.42	1.26, 1.37	1.29, 1.42	1.28, 1.41	1.28, 1.42	1.43	1.42
f	-	1.90	1.91	1.86	1.91	1.86	1.92	1.86	1.91	1.85	1.91	1.91	1.91	1.89	1.92
g	-	1.05	0.94	0.88	0.91	0.88	0.90	0.88	0.90	0.88	0.90	0.89	0.90	1.04	0.94
h	-	-	-	-	-	-	-	-	-	-	-	-	-	-	-

[a] in CD_3OD as solvent; [b] in $CDCl_3$ as solvent

Table S3. The ^{1}H NMR chemical shifts (ppm) of compounds **11-23**.

Protons	**11**[a]	**11**[b]	**12**[b]	**13**[b]	**14**[a]	**14**[b]	**15**[b]	**16**[b]	**17**[a]	**18**[b]	**19**[b]	**20**[b]	**21**[a]	**21**[b]	**22**[b]	**23**[b]
a	8.09	8.06	8.03	8.01	8.07	8.00	8.00	8.00	8.07	8.19	8.11	8.09	8.27	8.04	8.24	8.14
b	4.74	4.93	5,00	4.99	4.73	5.01	5.00	5.04	4.72	5.22	5.21	5.17	5.08	5.15	5.23	5.13
c	3.22	3.27	3.30	3.29	3.22	3.29	3.29	3.29	3.22	3.34	3.34	3.35	3.19	3.37	3.35	3.36
d	3.54	3.63	3.64	3.63	3.53	3.63	3.63	3.62	3.53	4.06	4.09	4.10	3.78	4.12	4.07	4.10
e	1.45	1.34	1.25, 1.34	1.25, 1.35	1.30, 1.41	1.34, 1.25	1.25, 1.34	1.25, 1.32	1.29	1.39	1.41	1.27, 1.40	1.30, 1.45	1.27, 1.40	1.39, 1.45	1.26, 1.40
f	1.90	1.80	1.79	1.78	1.90	1.78	1.79	1.78	1.91	1.83	1.83	1.82	1.93	1.79	1.82	1.81
g	1.04	0.88, 0.90	0.87	0.88	0.90	0.88	0.88	0.88	0.90	0.91	0.89	0.89	0.90	0.89	0.88	0.88
h	-	-	-	-	-	-	-	-	-	-	-	-	-	-	-	-

[a] in CD_3OD as solvent; [b] in $CDCl_3$ as solvent

Table S4. The ^{13}C NMR chemical shifts (ppm) of compounds **1c-10**.

Carbon	**1c**[b]	**2**[a]	**3**[a]	**3**[b]	**4**[a]	**4**[b]	**5**[a]	**5**[b]	**6**[a]	**6**[b]	**7**[a]	**8**[a]	**9**[a]	**10**[a]	**10**[b]
a	137.6	135.0	135.0	133.9	135.0	134.0	135.0	134.0	135.0	134.0	135.0	135.0	135.0	141.2	139.6
b	28.7	66.0	66.1	64.5	66.2	64.7	66.2	64.7	66.2	64.8	66.2	66.1	66.0	66.1	64.5
c	-	50.6	50.6	49.3	50.6	49.4	50.6	49.3	50.6	49.4	50.5	50.6	50.6	50.6	49.1
d	-	67.9	67.9	66.1	67.9	65.9	67.9	66.0	67.9	66.0	68.0	68.1	67.8	68.0	66.7
e, f	-	25.7, 20.8	32.5, 23.6, 23.7, 27.2	31.1, 26.0, 22.8, 22.3	32.9, 30.3, 27.5, 23.8, 23.7	31.5, 29.2, 29.0, 26.3, 22.9, 22.5	33.1, 30.7, 30.4, 27.5, 23.8	31.7, 29.3, 29.2, 29.1, 26.3, 22.9, 22.5	33.1, 30.8, 30.7, 30.5, 30.4, 27.5, 23.8	31.8, 29.5, 29.4, 29.3, 26.4, 23.0, 22.6	33.1, 30.8, 30.7, 30.6, 30.5, 30.4, 27.5, 23.8	33.1, 30.8, 30.7, 30.6, 30.5, 30.4, 27.5, 23.8	33.1, 30.8, 30.7, 30.5, 30.4, 27.5, 23.8	25.7, 20.9	24.4, 19.5
g	-	14.0	14.3	13.8	14.5	13.9	14.5	13.9	14.5	14.0	14.5	14.6	14.5	14.1	13.5

h	133.<br>6	131.<br>6	131.<br>6	130.<br>0	131.<br>6	130.<br>0	131.<br>6	130.<br>0	131.<br>6	130.<br>0	131.<br>6	131.<br>6	131.<br>6	131.<br>3	129.<br>3

[a] in CD_3OD as solvent; [b] in $CDCl_3$ as solvent

Table S5. The ^{13}C NMR chemical shifts (ppm) of compounds **11-23**.

Carbon	**11**[a]	**11**[b]	**12**[b]	**13**[b]	**14**[a]	**14**[b]	**15**[b]	**16**[b]	**17**[a]	**18**[b]	**19**[b]	**20**[b]	**21**[a]	**21**[b]	**22**[b]	**23**[b]
a	141.2	140	139.8	139.8	141.2	139.8	139.9	139.8	141.2	142.0	142.0	141.9	146.3	141.7	142.2	141.8
b	66.3	64.5	64.4	64.5	66.2	64.6	64.6	64.4	66.0	62.4	62.5	62.6	66.7	62.6	62.5	62.4
c	50.6	49.4	49.3	49.3	50.6	49.4	49.4	49.4	50.6	49.9	49.9	49.9	52.8	50.0	49.8	49.8
d	68.0	66.9	67.1	67.1	68.2	67.2	67.2	67.3	68.2	65.7	65.8	65.9	69.8	65.9	65.8	66.0
e, f	32.5, 27.2, 23.7, 23.6	31.3, 25.9, 22.7, 22.4	31.7, 29.2, 29.0, 26.2, 22.7, 22.5	31.8, 29.5, 29.4, 29.3, 29.2, 26.2, 22.7, 22.5	33.1, 30.8, 30.7, 30.5, 27.6, 23.8	31.8, 29.6, 29.4, 29.3, 22.8, 22.6	31.9, 29.6, 29.5, 29.3, 26.3, 22.8, 22.6	31.9, 29.7, 29.6, 29.5, 29.4, 29.3, 26.2, 25.9, 22.8, 22.6	33.2, 30.9, 30.8, 30.7, 30.6, 30.5, 27.6, 26.7, 23.8	31.4, 26.0, 23.0, 22.4	31.6, 27.5, 26.0, 23.4, 22.7	31.7, 29.2, 27.4, 26.4, 23.2, 22.6	35.6, 33.3, 33.2, 33.0, 30.0, 26.5, 26.3	31.9, 29.6, 29.5, 29.3, 26.4, 23.2, 22.6	29.7, 29.6, 29.5, 29.4, 27.1, 26.5, 25.7, 23.2, 22.7	31.9, 29.7, 29.6, 29.5, 29.3, 26.4
g	14.4	14.0	14.0	14.0	14.5	14.0	14.1	14.1	14.5	13.9	13.9	14.0	17.0	14.1	14.1	14.1
h	131.3	129.3	129.2	129.2	131.3	129.3	129.3	129.3	131.3	132.4	132.7	132.5	136.5	132.5	132.6	132.5

[a] in CD_3OD as solvent; [b] in $CDCl_3$ as solvent

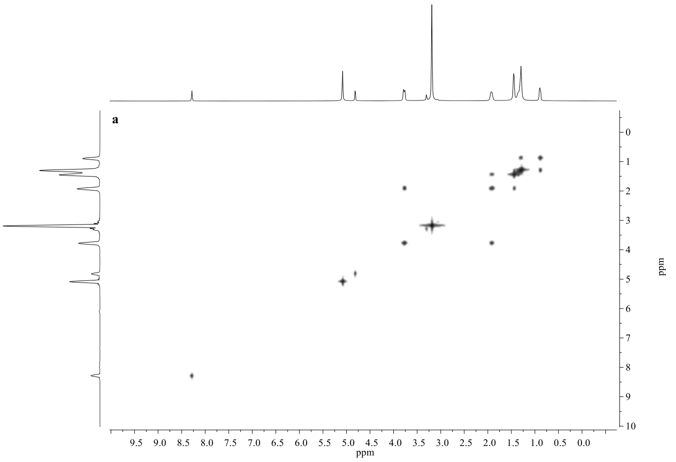

a)

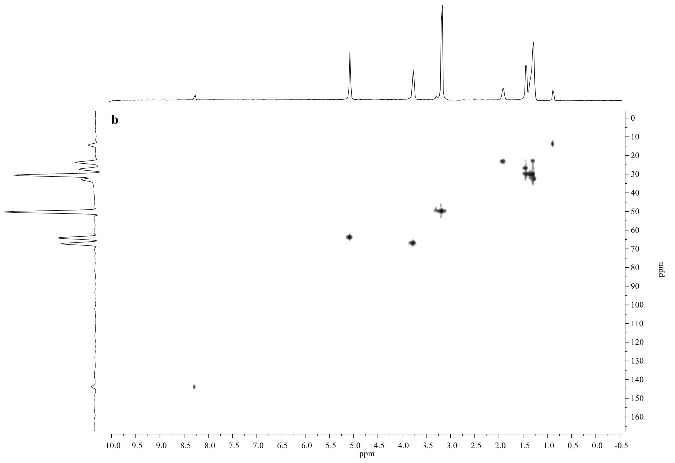

b)

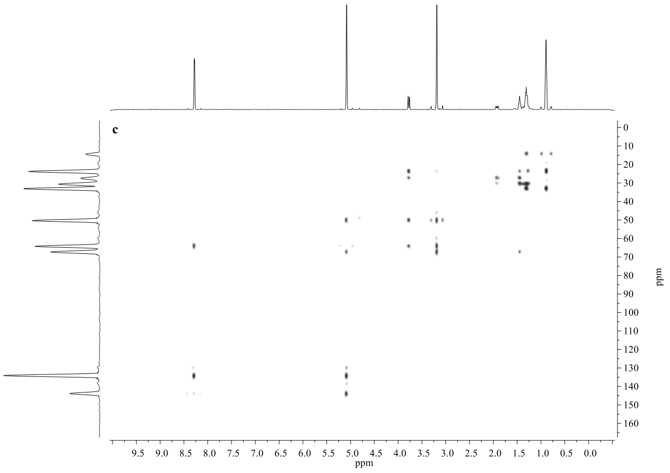

c)

Figure S1. 2D NMR spectra of compound **21**: COSY (a), HSQC (b) and HMBC (c).

Synthesis:

Synthesis*1,4-di-[N-(1-butyl)-N,N-dimethylammoniummethyl)benzene dibromide* (**2**) RT = 4 h, white solid (90%), m. p. 201-203 °C. ^{1}H NMR (CD_3OD) δ: 7.76 ppm (4H, a), 4.66 ppm (4H, b), 3.43 ppm (4H, d), 3.11 ppm (12H, c), 1.90 ppm (4H, f), 1.46 ppm (4H, e), 1.05 ppm (6H, g). ^{13}C NMR (CD_3OD) δ: 135.0 ppm (a), 131.6 ppm (h), 67.9 ppm (d), 66.0 ppm (b), 50.6 ppm (c), 25.7 and 20.8 ppm (e,f), 14.0 ppm (g). Elemental analysis for $C_{20}H_{38}N_2Br_2$ found (calc.): %N 5.93 (5.93); %C 50.43 (50.96); %H 8.44 (9.39). ESI(+)-MS (*m*/*z*): 153.1 ($C_{20}H_{38}N_2/2$). FT-IR (KBr) ν_{max}: 3503, 3014, 2967, 2874, 1618, 1489, 1384, 1221, 1011, 942, 884, 826, 733.

1,4-di-[N-(1-hexyl)-N,N-dimethylammoniummethyl)benzene dibromide (**3**) RT = 6 h, white solid (80%), m. p. 206-207 °C. ^{1}H NMR (CD_3OD) δ: 7.76 ppm (4H, a), 4.66 ppm (4H, b), 3.41 ppm (4H, d), 3.11 ppm (12H, c), 1.91 ppm (4H, f), 1.41 ppm (12H, e), 0.94 ppm (6H, g). ^{1}H NMR ($CDCl_3$) δ: 7.86 ppm (4H, a), 5.28 ppm (4H, b), 3.61 ppm (4H, d), 3.27 ppm (12H, c), 1.86 ppm (4H, f), 1.37 ppm (12H, e), 0.88 ppm (6H, g). ^{13}C NMR (CD_3OD) δ: 135.0 ppm (a), 131.6 ppm (h), 67.9 ppm (d), 66.1 ppm (b), 50.6 ppm (c), 32.5, 27.2, 23.7 and 23.6 ppm (e,f), 14.3 ppm (g). ^{13}C NMR ($CDCl_3$) δ: 133.9 ppm (a), 130.0 ppm (h), 66.1 ppm (d), 64.5 ppm (b), 49.3 ppm (c), 31.1, 26.0, 22.8 and 22.3 ppm (e,f), 13.8 ppm (g). Elemental analysis for $C_{24}H_{46}N_2Br_2 \cdot 0.5\ H_2O$ found (calc.): %N 5.34 (5.27); %C 54.19 (54.24); %H 9.12 (8.91). ESI(+)-MS (*m*/*z*): 181.2 ($C_{24}H_{46}N_2/2$).

1,4-di-[N,N-dimethyl-N-(1-octyl)ammoniummethyl)benzene dibromide (**4**) RT = 8 h, white solid (90%), m. p. 210-211 °C. ^{1}H NMR (CD_3OD) δ: 7.75 ppm (4H, a), 4.64 ppm (4H, b), 3.40 ppm (4H, d), 3.10 ppm (12H, c), 1.91 ppm (4H, f), 1.41 and 1.33 ppm (20H, e), 0.91 ppm (6H, g). ^{1}H NMR ($CDCl_3$) δ: 7.84 ppm (4H, a), 5.29 ppm (4H, b), 3.60 ppm (4H, d), 3.26 ppm (12H, c), 1.86 ppm (4H, f), 1.27, 1.37 ppm (20H, e), 0.88 ppm (6H, g). ^{13}C NMR (CD_3OD) δ: 135.0 ppm (a), 131.6 ppm (h), 67.9 ppm (d), 66.2 ppm (b), 50.6 ppm (c), 32.9, 30.3, 27.5, 23.7 and 23.7 ppm (e,f), 14.5 ppm (g). ^{13}C NMR ($CDCl_3$) δ: 134.0 ppm (a), 130.0 ppm (h), 65.9 ppm (d), 64.7 ppm (b), 49.4 ppm (c), 31.5, 29.2, 29.0, 26.3, 22.9 and 22.5 ppm (e,f), 13.9 ppm (g). Elemental analysis for $C_{28}H_{54}N_2Br_2$ found (calc.): %N 4.99 (4.84); %C 58.26 (58.13); %H 9.60 (9.41). ESI(+)-MS (*m*/*z*): 209.2 ($C_{28}H_{54}N_2/2$).

1,4-di-[N-(1-decyl)-N,N-dimethylammoniummethyl)benzene dibromide (**5**) RT = 8 h, white solid (90%), m. p. 216-217 °C. ^{1}H NMR (CD_3OD) δ: 7.75 ppm (4H, a), 4.65 ppm (4H, b), 3.40 ppm (4H, d), 3.10 ppm (12H,

c), 1.92 ppm (4H, f), 1.41 and 1.30 ppm (28H, e), 0.90 ppm (6H, g). ^{1}H NMR ($CDCl_3$) δ: 7.84 ppm (4H, a), 5.29 ppm (4H, b), 3.60 ppm (4H, d), 3.26 ppm (12H, c), 1.86 ppm (4H, f), 1.37 and 1.26 ppm (28H, e), 0.88 ppm (6H, g). ^{13}C NMR (CD_3OD) δ: 135.0 ppm (a), 131.6 ppm (h), 67.9 ppm (d), 66.2 ppm (b), 50.6 ppm (c), 33.1, 30.7, 30.4, 27.5 and 23.8 ppm (e,f), 14.5 ppm (g). ^{13}C NMR ($CDCl_3$) δ: 134.0 ppm (a), 130.0 ppm (h), 66.0 ppm (d), 64.7 ppm (b), 49.3 ppm (c), 31.7, 29.3, 29.2, 29.1, 26.3, 22.9 and 22.5 ppm (e,f), 13.9 ppm (g). Elemental analysis for $C_{32}H_{62}N_2Br_2$ found (calc.): %N 4.54 (4.41); %C 60.33 (60.56); %H 10.03 (9.85). ESI(+)-MS (*m/z*): 237.2 ($C_{32}H_{62}N_2/2$).

1,4-di-[N-(1-dodecyl)-N,N-dimethylammoniummethyl)benzene dibromide (**6**) RT = 9 h, white solid (95%), m. p. 219-220 °C. ^{1}H NMR (CD_3OD) δ: 7.75 ppm (4H, a), 4.64 ppm (4H, b), 3.39 ppm (4H, d), 3.10 ppm (12H, c), 1.91 ppm (4H, f), 1.42 and 1.28 ppm (36H, e), 0.90 ppm (6H, g). ^{1}H NMR ($CDCl_3$) δ: 7.82 ppm (4H, a), 5.28 ppm (4H, b), 3.59 ppm (4H, d), 3.25 ppm (12H, c), 1.85 ppm (4H, f), 1.37 and 1.26 ppm (36H, e), 0.88 ppm (6H, g). ^{13}C NMR (CD_3OD) δ: 135.0 ppm (a), 131.6 ppm (h), 67.9 ppm (d), 66.2 ppm (b), 50.6 ppm (c), 33.1, 30.8, 30.7, 30.5, 30.4, 27.5 and 23.8 ppm (e,f), 14.5 ppm (g). ^{13}C NMR ($CDCl_3$) δ: 134.0 ppm (a), 130.0 ppm (h), 66.0 ppm (d), 64.8 ppm (b), 49.4 ppm (c), 31.8, 29.5, 29.4, 29.3, 26.4, 23.0 and 22.6 ppm (e,f), 14.0 ppm (g). Elemental analysis for $C_{36}H_{70}N_2Br_2$ found (calc.): %N 4.28 (4.06); %C 62.34 (62.61); %H 10.39 (10.21). ESI(+)-MS (*m/z*): 265.7 ($C_{36}H_{70}N_2/2$). FT-IR (KBr) ν_{max}: 3402, 3009, 2959, 2918, 2854, 1490, 1456, 1430, 1219, 1005, 880, 824, 721.

1,4-di-[N,N-dimethyl-N-(1-tetradecyl)ammoniummethyl)benzene dibromide (**7**) RT = 9 h, white solid (95%), m. p. 221-222 °C. ^{1}H NMR (CD_3OD) δ: 7.75 ppm (4H, a), 4.64 ppm (4H, b), 3.40 ppm (4H, d), 3.10 ppm (12H, c), 1.91 ppm (4H, f), 1.42 and 1.29 ppm (44H, e), 0.90 ppm (6H, g). ^{13}C NMR (CD_3OD) δ: 135.0 ppm (a), 131.6 ppm (h), 68.0 ppm (d), 66.2 ppm (b), 50.5 ppm (c), 33.1, 30.8, 30.7, 30.6, 30.5, 30.4, 27.5 and 23.8 ppm (e,f), 14.5 ppm (g). Elemental analysis for $C_{40}H_{78}N_2Br_2$ found (calc.): %N 3.66 (3.75); %C 64.22 (64.33); %H 10.69 (10.53). ESI(+)-MS (*m/z*): 293.5 ($C_{40}H_{78}N_2/2$).

1,4-di-[N-(1-hexadecyl)-N,N-dimethylammoniummethyl)benzene dibromide (**8**) RT = 10 h, white solid (90%), m. p. 223-224 °C. ^{1}H NMR (CD_3OD) δ: 7.75 ppm (4H, a), 4.64 ppm (4H, b), 3.38 ppm (4H, d), 3.09 ppm (12H, c), 1.91 ppm (4H, f), 1.41 and 1.28 ppm (52H, e), 0.89 ppm (6H, g). ^{13}C NMR (CD_3OD) δ: 135.0 ppm (a), 131.6 ppm (h), 68.1 ppm (d), 66.1 ppm (b), 50.6 ppm (c), 33.1, 30.8, 30.7, 30.6, 30.5, 30.4, 27.5 and 23.8 ppm (e,f), 14.6 ppm (g). Elemental analysis for $C_{44}H_{86}N_2Br_2$ found (calc.): %N 3.56 (3.49); %C 65.76 (65.81); %H 10.98 (10.79). ESI(+)-MS (*m/z*): 321.6 ($C_{44}H_{86}N_2/2$).

1,4-di-[N,N-dimethyl-N-(1-octadecyl)ammoniummethyl)benzene dibromide (**9**) RT = 10 h, white solid (90%), m. p. 224-225 °C. ^{1}H NMR (CD_3OD) δ: 7.74 ppm (4H, a), 4.64 ppm (4H, b), 3.38 ppm (4H, d), 3.10 ppm (12H, c), 1.91 ppm (4H, f), 1.42 and 1.28 ppm (60H, e), 0.90 ppm (6H, g). ^{13}C NMR (CD_3OD) δ: 135.0 ppm (a), 131.6 ppm (h), 67.8 ppm (d), 66.0 ppm (b), 50.6 ppm (c), 33.1, 30.8, 30.7, 30.6, 30.5, 30.4, 27.5 and 23.8 ppm (e,f), 14.5 ppm (g). Elemental analysis for $C_{48}H_{94}N_2Br_2$ found (calc.): %N 3.40 (3.26); %C 67.43 (67.11); %H 11.26 (11.03). ESI(+)-MS (*m/z*): 349.8 ($C_{48}H_{94}N_2/2$). FT-IR (KBr) ν_{max}: 3383, 3025, 2921, 2851, 1487, 1420, 1384, 1221, 1011, 872, 826, 722.

1,3,5-tris-[N-(1-butyl)-N,N-dimethylammoniummethyl)benzene tribromide (**10**) RT = 4 h, white solid (50%), m. p. 218-219 °C. ^{1}H NMR (CD_3OD) δ: 8.09 ppm (3H, a), 4.73 ppm (6H, b), 3.54 ppm (6H, d), 3.22 ppm (18H, c), 1.89 ppm (6H, f), 1.43 ppm (6H, e), 1.04 ppm (9H, g). ^{1}H NMR ($CDCl_3$): 8.01 ppm (3H, a), 4.83 ppm (6H, b), 3.42 ppm (6H, d), 3.11 ppm (18H, c), 1.81 ppm (6H, f), 1.42 ppm (6H, e), 0.94 ppm (9H, g). ^{13}C NMR (CD_3OD) δ: 141.2 ppm (a), 131.3 ppm (h), 68.0 ppm (d), 66.1 ppm (b), 50.6 ppm (c), 25.7 and 20.9 ppm (e,f), 14.1 ppm (g). ^{13}C NMR ($CDCl_3$) δ: 139.6 ppm (a), 129.3 ppm (h), 66.7 ppm (d), 64.5 ppm (b), 49.1 ppm (c), 24.4 ppm (f), 19.5 ppm (e), 13.5 ppm (g). Elemental analysis for $C_{27}H_{54}N_3Br_3 \cdot H_2O$ found (calc.): %N 5.76 (6.19); %C 47.33 (47.80); %H 8.53 (8.32). ESI(+)-MS (*m/z*): 140.2 ($C_{27}H_{54}N_3/3$). FT-IR (KBr) ν_{max}: 3434, 3014, 2983, 2878, 2076, 1642, 1488, 1454, 1384, 1190, 1038, 882, 754.

1,3,5-tris-[N-(1-hexyl)-N,N-dimethylammoniummethyl)benzene tribromide (**11**) RT = 6 h, white solid (50%), m. p. 221-222 °C. ^{1}H NMR (CD_3OD) δ: 8.09 ppm (3H, a), 4.74 ppm (6H, b), 3.54 ppm (6H, d), 3.22 ppm (18H, c), 1.90 ppm (6H, f), 1.45 ppm (18H, e), 1.04 ppm (9H, g). ^{1}H NMR ($CDCl_3$): 8.06 ppm (3H, a), 4.93 ppm (6H, b), 3.63 ppm (6H, d), 3.27 ppm (18H, c), 1.80 ppm (6H, f), 1.34 ppm (18H, e), 0.90 and 0.88 ppm (9H, g). ^{13}C NMR (CD_3OD) δ: 141.2 ppm (a), 131.3 ppm (h), 68.0 ppm (d), 66.3 ppm (b), 50.6 ppm (c), 32.5, 27.2, 23.7 and 23.6 ppm (e,f), 14.4 ppm (g). ^{13}C NMR ($CDCl_3$) δ: 140.0 ppm (a), 129.3 ppm (h), 66.9 ppm (d), 64.5 ppm (b), 49.4 ppm (c), 31.3, 29.9, 22.7 and 22.4 ppm (e, f), 14.0 ppm (g). Elemental analysis for $C_{33}H_{66}N_3Br_3 \cdot 1.5\ H_2O$ found (calc.): %N 5.25 (5.45); %C 51.64 (51.37); %H 9.22 (9.01). ESI(+)-MS (*m/z*): 168.3 ($C_{33}H_{66}N_3/3$).

1,3,5-tris-[N,N-dimethyl-N-(1-octyl)ammoniummethyl)benzene tribromide (**12**) RT = 7 h, white solid (60%), m. p. 223-224 °C. ^{1}H NMR ($CDCl_3$) δ: 8.03 ppm (3H, a), 5.00 ppm (6H, b), 3.64 ppm (6H, d), 3.30 ppm (18H, c), 1.79 ppm (6H, f), 1.34 and 1.25 ppm (30H, e), 0.87 ppm (9H, g). ^{13}C NMR ($CDCl_3$) δ: 139.8 ppm (a), 129.2 ppm (h), 67.1 ppm (d), 64.4 ppm (b), 49.3 ppm (c), 31.7, 29.2, 29.0, 26.2, 22.7 and 22.5 ppm (e,f), 14.0 ppm (g). Elemental analysis for $C_{39}H_{78}N_3Br_3 \cdot H_2O$ found (calc.): %N 4.69 (4.96); %C 55.41 (55.32); %H 9.78 (9.52). ESI(+)-MS (*m/z*): 196.4 ($C_{39}H_{78}N_3/3$).

1,3,5-tris-[N-(1-decyl)-N,N-dimethylammoniummethyl)benzene tribromide (**13**) RT = 8 h, white solid (40%), m. p. 225-226 °C. ^{1}H NMR ($CDCl_3$) δ: 8.01 ppm (3H, a), 4.99 ppm (6H, b), 3.63 ppm (6H, d), 3.29 ppm (18H, c), 1.78 ppm (6H, f), 1.35 and 1.25 ppm (42H, e), 0.88 ppm (9H, g). ^{13}C NMR ($CDCl_3$) δ: 139.8 ppm (a), 129.9 ppm (h), 67.1 ppm (d), 64.5 ppm (b), 49.3 ppm (c), 31.8, 29.5, 29.4, 29.3, 29.2, 26.2, 22.7 and 22.5 ppm (e,f), 14.0 ppm (g). Elemental analysis for $C_{45}H_{90}N_3Br_3 \cdot H_2O$ found (calc.): %N 4.28 (4.51); %C 58.39 (58.06); %H 10.14 (9.96). ESI(+)-MS (*m/z*): 224.2 ($C_{45}H_{90}N_3/3$).

1,3,5-tris-[N-(1-dodecyl)-N,N-dimethylammoniummethyl)benzene tribromide (**14**) RT = 10 h, white solid (70%), m. p. 227-228 °C. ^{1}H NMR (CD_3OD) δ: 8.07 ppm (3H, a), 4.73 ppm (6H, b), 3.53 ppm (6H, d), 3.22 ppm (18H, c), 1.90 ppm (6H, f), 1.41 and 1.30 ppm (54H, e), 0.90 ppm (9H, g). ^{1}H NMR ($CDCl_3$) δ: 8.00 ppm (3H, a), 5.01 ppm (6H, b), 3.63 ppm (6H, d), 3.29 ppm (18H, c), 1.78 ppm (6H, f), 1.34 and 1.25 ppm (54H, e), 0.88 ppm (9H, g). ^{13}C NMR (CD_3OD) δ: 141.2 ppm (a), 131.3 ppm (h), 68.2 ppm (d), 66.2 ppm (b), 50.6 ppm (c), 33.1, 30.8, 30.7, 30.5, 27.6 and 23.8 ppm (e,f), 14.5 ppm (g). ^{13}C NMR ($CDCl_3$) δ: 139.8 ppm (a), 129.3 ppm (h), 67.2 ppm (d), 64.6 ppm (b), 49.4 ppm (c), 31.8, 29.6, 29.4, 29.3, 22.8 and 22.6 ppm (e,f), 14.0 ppm (g). Elemental analysis for $C_{51}H_{102}N_3Br_3 \cdot 2\ H_2O$ found (calc.): %N 4.03 (4.07); %C 59.06 (59.29); %H 10.38 (10.34). ESI(+)-MS (*m/z*): 252.5 ($C_{51}H_{102}N_3/3$). FT-IR (KBr) ν_{max}: 3437, 3018, 2928, 2854, 1755, 1490, 1468, 1436, 1381, 1195, 1033, 753, 724.

1,3,5-tris-[N,N-dimethyl-N-(1-tetradecyl)ammoniummethyl)benzene tribromide (**15**) RT = 12 h, white solid (60%), m. p. 229-230 °C. ^{1}H NMR ($CDCl_3$) δ: 8.00 ppm (3H, a), 5.00 ppm (6H, b), 3.63 ppm (6H, d), 3.29 ppm (18H, c), 1.79 ppm (6H, f), 1.34 and 1.25 ppm (66H, e), 0.88 ppm (9H, g). ^{13}C NMR ($CDCl_3$) δ: 139.9 ppm (a), 129.3 ppm (h), 67.2 ppm (d), 64.6 ppm (b), 49.4 ppm (c), 31.9, 29.6, 29.5, 29.3, 26.3, 22.8 and 22.6 ppm (e,f), 14.1 ppm (g). Elemental analysis for $C_{57}H_{114}N_3Br_3 \cdot 2\ H_2O$ found (calc.): %N 3.63 (3.76); %C 61.01 (61.28); %H 10.59 (10.64). ESI(+)-MS (*m/z*): 280.5 ($C_{57}H_{114}N_3/3$). FT-IR (KBr) ν_{max}: 3435, 3018, 2920, 2853, 1620, 1489, 1469, 1432, 1373, 1197, 1030, 897, 753, 722, 672.

1,3,5-tris-[N-(1-hexadecyl)-N,N-dimethylammoniummethyl)benzene tribromide (**16**) RT = 13 h, white solid (50%), m. p. 232-234 °C. ^{1}H NMR ($CDCl_3$) δ: 8.00 ppm (3H, a), 5.04 ppm (6H, b), 3.62 ppm (6H, d), 3.29 ppm (18H, c), 1.78 ppm (6H, f), 1.32 and 1.25 ppm (78H, e), 0.88 ppm (9H, g). ^{13}C NMR ($CDCl_3$) δ: 139.8 ppm (a), 129.3 ppm (h), 67.3 ppm (d), 64.4 ppm (b), 49.4 ppm (c), 31.9, 29.7, 29.6, 29.5, 29.4, 29.3, 26.2, 25.9, 22.8 and 22.6 ppm (e,f), 14.1 ppm (g). Elemental analysis for $C_{63}H_{126}N_3Br_3 \cdot 1.5\ H_2O$ found (calc.): %N 3.34 (3.52); %C 64.20 (63.46); %H 11.10 (10.90). ESI(+)-MS (*m/z*): 308.6 ($C_{63}H_{126}N_3/3$).

1,3,5-tris-[N,N-dimethyl-N-(1-octadecyl)ammoniummethyl)benzene tribromide (**17**) RT = 15 h, white solid (85%), m. p. 235-236 °C. ^{1}H NMR (CD_3OD) δ: 8.07 ppm (3H, a), 4.72 ppm (6H, b), 3.53 ppm (6H, d),

3.22 ppm (18H, c), 1.91 ppm (6H, f), 1.29 ppm (90H, e), 0.90 ppm (9H, g). ^{13}C NMR (CD_3OD) δ: 141.2 ppm (a), 131.3 ppm (h), 68.2 ppm (d), 66.0 ppm (b), 50.6 ppm (c), 33.2, 30.9, 30.8, 30.7, 30.6, 30.5, 27.6, 26.7 and 23.8 ppm (e,f), 14.5 ppm (g). Elemental analysis for $C_{69}H_{138}N_3Br_3 \cdot H_2O$ found (calc.): %N 3.23 (3.31); %C 65.45 (65.38); %H 11.29 (11.13). ESI(+)-MS (*m/z*): 336.6 ($C_{69}H_{138}N_3/3$). FT-IR (KBr) ν_{max}: 3426, 3012, 2921, 2852, 1717, 1625, 1465, 1453, 1373, 1190, 1030, 904, 755, 720.

1,2,4,5-tetrakis-[N-(1-hexyl)-N,N-dimethylammoniummethyl)benzene tetrabromide (**18**) RT = 7 h, white solid (15%), m. p. 217-219 °C. ^1H NMR ($CDCl_3$) δ: 8.19 ppm (2H, a), 5.22 ppm (8H, b), 4.06 ppm (8H, d), 3.34 ppm (24H, c), 1.83 ppm (8H, f), 1.39 ppm (24H, e), 0.91 ppm (12H, g). ^{13}C NMR ($CDCl_3$) δ: 142.0 ppm (a), 132.4 ppm (h), 65.7 ppm (d), 62.4 ppm (b), 49.9 ppm (c), 31.4, 26.0, 23.0 and 22.4 ppm (e,f), 13.9 ppm (g). Elemental analysis for $C_{42}H_{86}N_4Br_4 \cdot H_2O$ found (calc.): %N 5.51 (5.69); %C 51.36 (51.22); %H 9.34 (9.01). ESI(+)-MS (*m/z*): 161.8 ($C_{42}H_{86}N_4/4$).

1,2,4,5-tetrakis-[N,N-dimethyl-N-(1-octyl)ammoniummethyl)benzene tetrabromide (**19**) RT = 9 h, white solid (5%), m. p. 220-221 °C. ^1H NMR ($CDCl_3$) δ: 8.11 ppm (2H, a), 5.21 ppm (8H, b), 4.09 ppm (8H, d), 3.34 ppm (24H, c), 1.83 ppm (8H, f), 1.41 ppm (40H, e), 0.89 ppm (12H, g). ^{13}C NMR ($CDCl_3$) δ: 142.0 ppm (a), 132.7 ppm (h), 65.8 ppm (d), 62.5 ppm (b), 49.9 ppm (c), 31.6, 27.5, 26.0, 23.4 and 22.7 ppm (e,f), 13.9 ppm (g). Elemental analysis for $C_{50}H_{102}N_4Br_4 \cdot 2\ H_2O$ found (calc.): %N 4.69 (5.02); %C 54.69 (53.86); %H 9.83 (9.58). ESI(+)-MS (*m/z*): 189.8 ($C_{50}H_{102}N_4/4$).

1,2,4,5-tetrakis-[N-(1-decyl)-N,N-dimethylammoniummethyl)benzene tetrabromide (**20**) RT = 10 h, white solid (30%), m. p. 221-223 °C. ^1H NMR ($CDCl_3$) δ: 8.09 ppm (2H, a), 5.17 ppm (8H, b), 4.10 ppm (8H, d), 3.35 ppm (24H, c), 1.82 ppm (8H, f), 1.40 and 1.27 ppm (56H, e), 0.89 ppm (12H, g). ^{13}C NMR ($CDCl_3$) δ: 141.9 ppm (a), 132.5 ppm (h), 65.9 ppm (d), 62.6 ppm (b), 49.9 ppm (c), 31.7, 29.2, 27.4, 26.4, 23.2 and 22.6 ppm (e,f), 14.0 ppm (g). Elemental analysis for $C_{58}H_{118}N_4Br_4 \cdot H_2O$ found (calc.): %N 4.47 (4.63); %C 57.34 (57.61); %H 10.05 (10.00). ESI(+)-MS (*m/z*): 217.9 ($C_{58}H_{118}N_4/4$).

1,2,4,5-tetrakis-[N-(1-dodecyl)-N,N-dimethylammoniummethyl)benzene tetrabromide (**21**) RT = 12 h, white solid (70%), m. p. 224-226 °C. ^1H NMR (CD_3OD) δ: 8.27 ppm (2H, a), 5.08 ppm (8H, b), 3.78 ppm (8H, d), 3.19 ppm (24H, c), 1.93 ppm (8H, f), 1.45 and 1.30 ppm (72H, e), 0.90 ppm (12H, g). ^1H NMR ($CDCl_3$) δ: 8.04 ppm (2H, a), 5.15 ppm (8H, b), 4.12 ppm (8H, d), 3.37 ppm (24H, c), 1.79 ppm (8H, f), 1.40 and 1.27 ppm (72H, e), 0.89 ppm (12H, g). ^{13}C NMR (CD_3OD) δ: 146.3 ppm (a), 136.5 ppm (h), 69.8 ppm (d), 66.7 ppm (b), 52.8 ppm (c), 35.6, 33.3, 33.2, 33.0, 30.0, 26.5 and 26.3 ppm (e,f), 17.0 ppm (g). ^{13}C NMR ($CDCl_3$) δ: 141.7 ppm (a), 132.5 ppm (h), 65.9 ppm (d), 62.6 ppm (b), 50.0 ppm (c), 31.9, 29.6, 29.5, 29.3, 26.4, 23.2 and 22.6 ppm (e,f), 14.1 ppm (g). Elemental analysis for $C_{66}H_{134}N_4Br_4 \cdot 2\ H_2O$ found (calc.): %N 4.24 (4.18); %C 59.22 (59.18); %H 10.52 (10.38). ESI(+)-MS (*m/z*): 245.9 ($C_{66}H_{134}N_4/4$). FT-IR (KBr) ν_{max}: 3409, 3020, 2924, 2854, 1620, 1469, 1380, 1235, 1015, 891, 866, 849, 722.

1,2,4,5-tetrakis-[N,N-dimethyl-N-(1-tetradecyl)ammoniummethyl)benzene tetrabromide (**22**) RT = 13 h, white solid (50%), m. p. 225-226 °C. ^1H NMR ($CDCl_3$) δ: 8.24 ppm (2H, a), 5.23 ppm (8H, b), 4.07 ppm (8H, d), 3.35 ppm (24H, c), 1.82 ppm (8H, f), 1.45 and 1.39 ppm (88H, e), 0.88 ppm (12H, g). ^{13}C NMR ($CDCl_3$) δ: 142.2 ppm (a), 132.6 ppm (h), 65.8 ppm (d), 62.5 ppm (b), 49.8 ppm (c), 29.7, 29.6, 29.5, 29.4, 27.1, 26.5, 25.7, 23.2 and 22.7 ppm (e,f), 14.1 ppm (g). Elemental analysis for $C_{74}H_{150}N_4Br_4 \cdot H_2O$ found (calc.): %N 4.13 (3.91); %C 61.65 (62.00); %H 10.62 (10.69). ESI(+)-MS (*m/z*): 273.9 ($C_{74}H_{150}N_4/4$). FT-IR (KBr) ν_{max}: 3020, 2924, 2854, 1620, 1469, 1380, 1235, 1015, 891, 866, 849, 722.

1,2,4,5-tetrakis-[N-(1-hexadecyl)-N,N-dimethylammoniummethyl)benzene tetrabromide (**23**) RT = 14 h, white solid (62%), m. p. 228-230 °C. ^1H NMR ($CDCl_3$) δ: 8.14 ppm (2H, a), 5.14 ppm (8H, b), 4.10 ppm (8H, d), 3.36 ppm (24H, c), 1.81 ppm (8H, f), 1.40 and 1.26 ppm (104H, e), 0.88 ppm (12H, g). ^{13}C NMR ($CDCl_3$) δ: 141.8 ppm (a), 132.5 ppm (h), 66.0 ppm (d), 62.4 ppm (b), 49.8 ppm (c), 31.9, 29.7, 29.6, 29.5, 29.3 and 26.4 ppm (e,f), 14.1 ppm (g). Elemental analysis for $C_{82}H_{166}N_4Br_4 \cdot H_2O$ found (calc.): %N 3.73 (3.62);

%C 63.46 (63.71); %H 10.73 (10.95). ESI(+)-MS (*m*/*z*): 301.9 ($C_{82}H_{166}N_4$/4). FT-IR (KBr) ν_{max}: 3436, 3010, 2919, 2851, 1622, 1468, 1380, 1255, 1002, 722.
